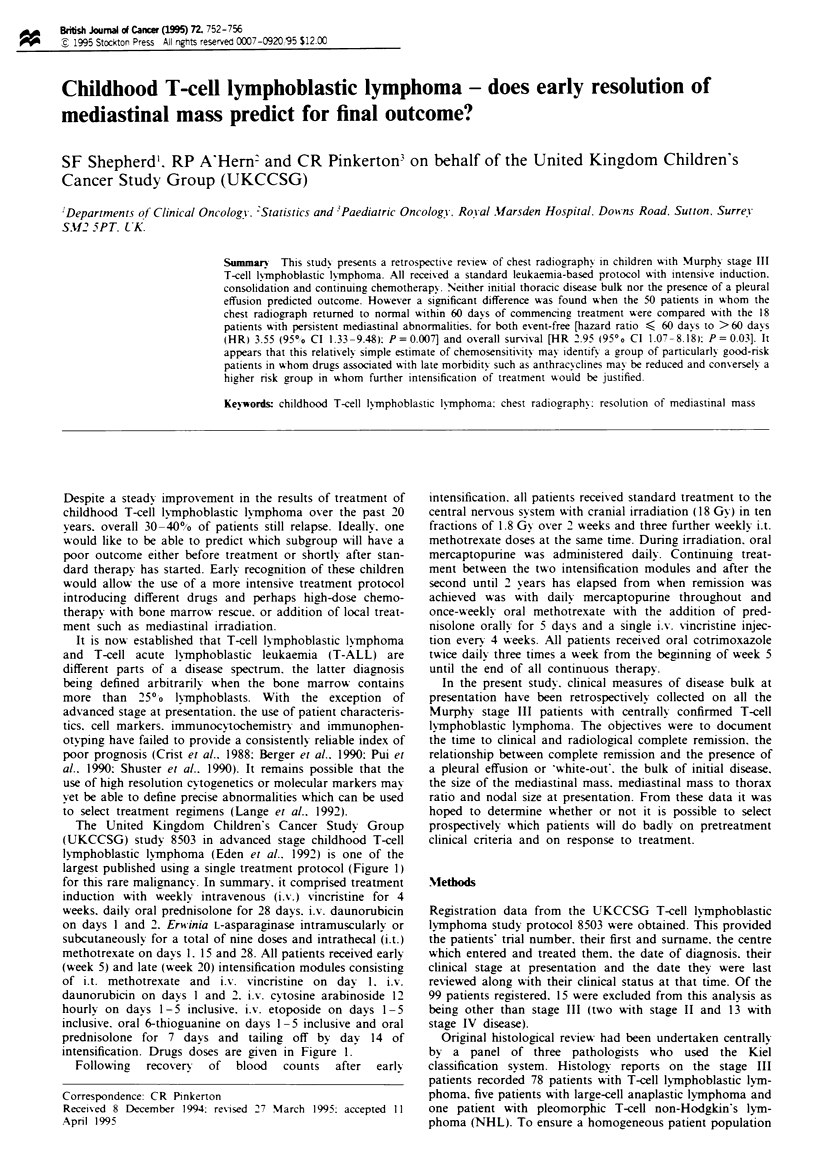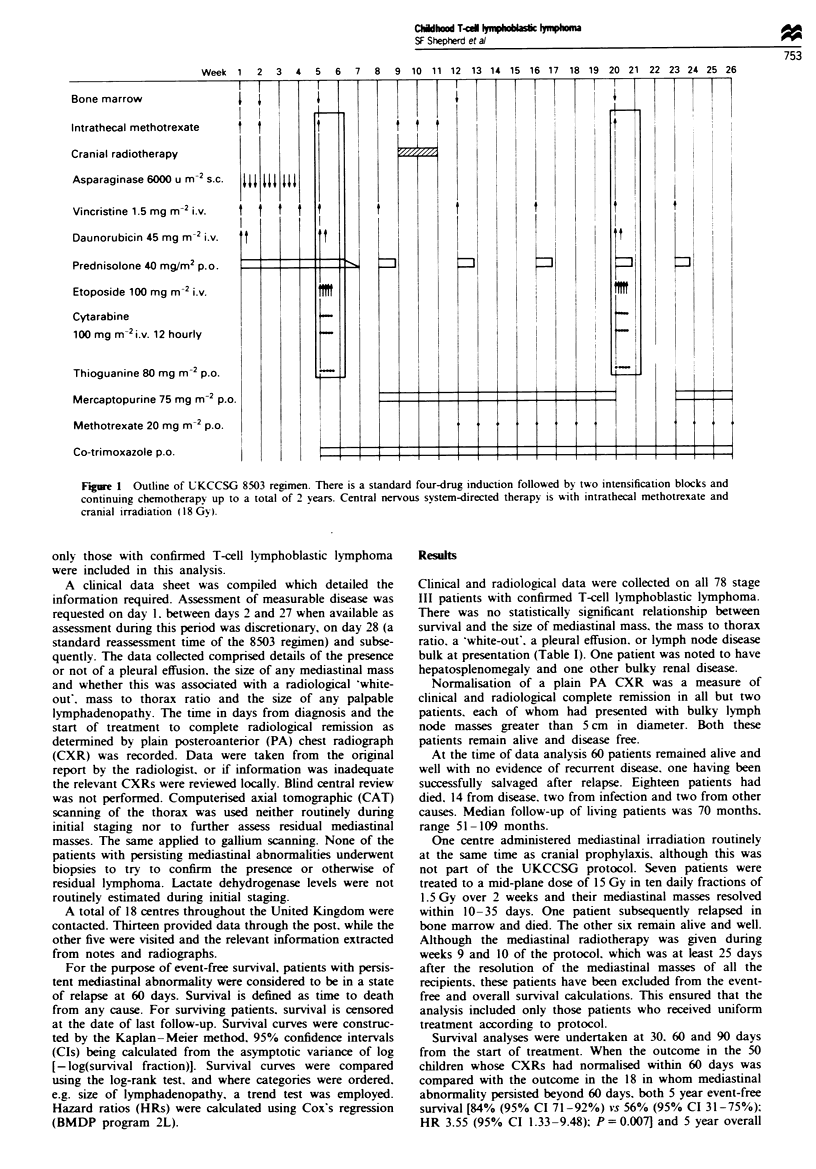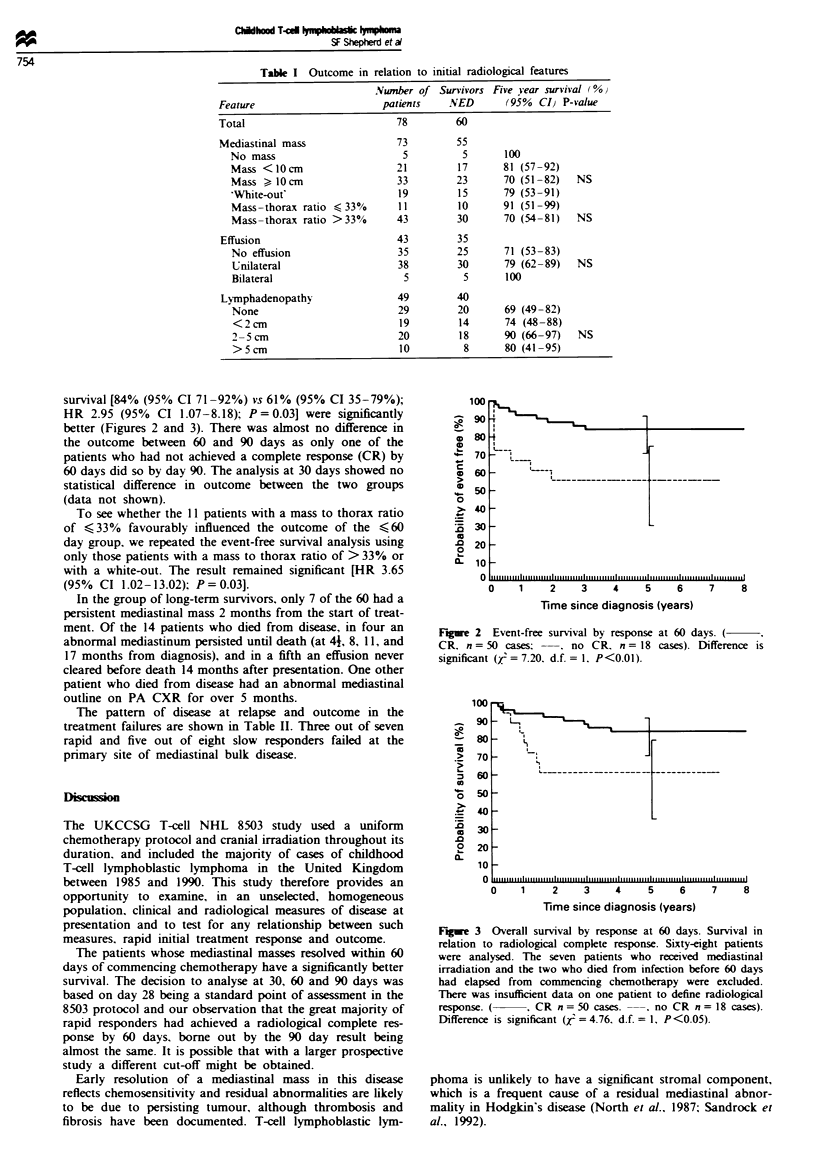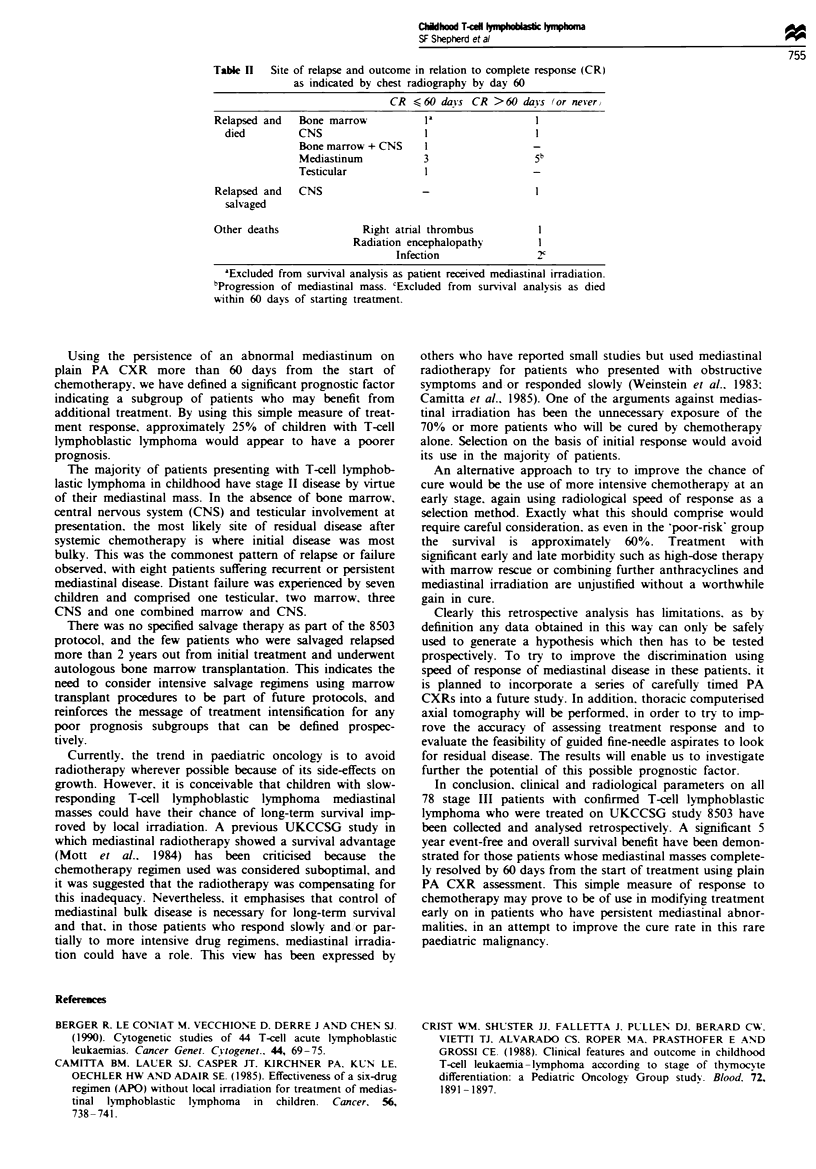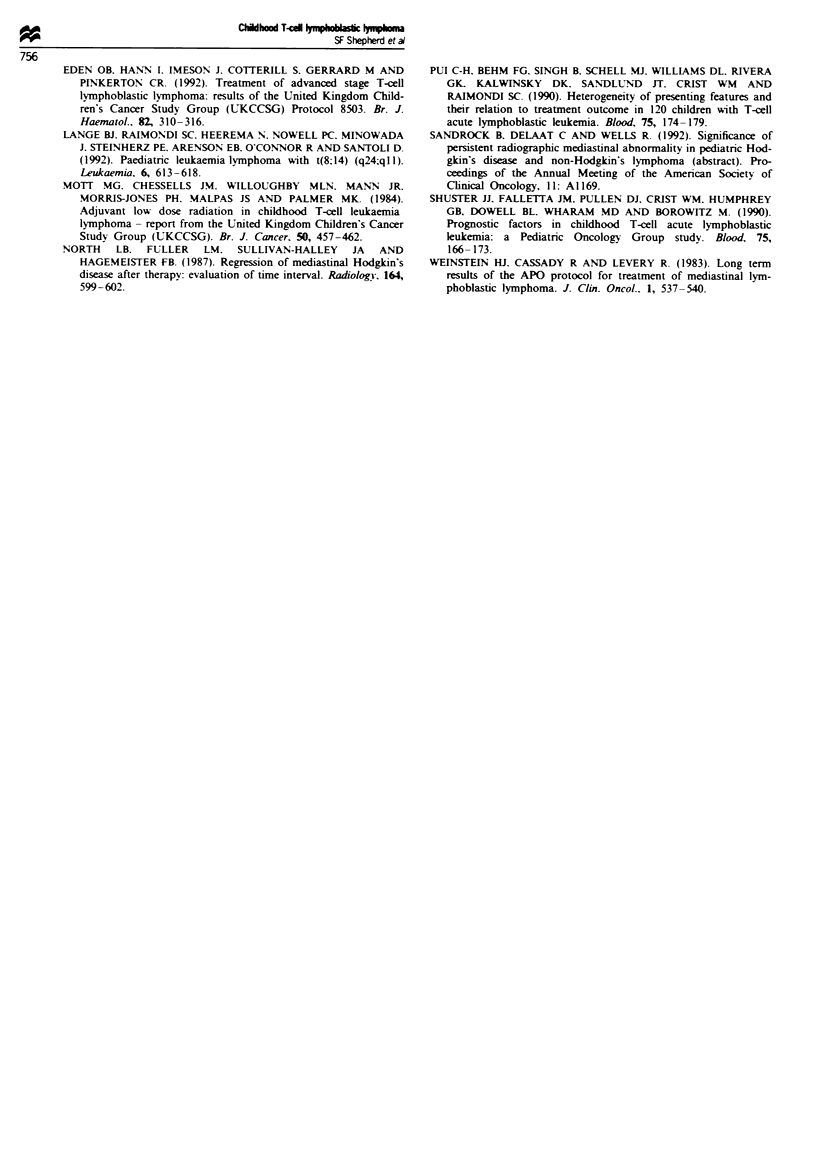# Childhood T-cell lymphoblastic lymphoma--does early resolution of mediastinal mass predict for final outcome? The United Kingdom Children's Cancer Study Group (UKCCSG).

**DOI:** 10.1038/bjc.1995.405

**Published:** 1995-09

**Authors:** S. F. Shepherd, R. P. A'Hern, C. R. Pinkerton

**Affiliations:** Department of Clinical Oncology, Royal Marsden Hospital, Surrey, UK.

## Abstract

This study presents a retrospective review of chest radiography in children with Murphy stage III T-cell lymphoblastic lymphoma. All received a standard leukaemia-based protocol with intensive induction, consolidation and continuing chemotherapy. Neither initial thoracic disease bulk nor the presence of a pleural effusion predicted outcome. However a significant difference was found when the 50 patients in whom the chest radiograph returned to normal within 60 days of commencing treatment were compared with the 18 patients with persistent mediastinal abnormalities, for both event-free [hazard ratio < or = 60 days to > 60 days (HR) 3.55 (95% CI 1.33-9.48); P = 0.007] and overall survival [HR 2.95 (95% CI 1.07-8.18); P = 0.03]. It appears that this relatively simple estimate of chemosensitivity may identify a group of particularly good-risk patients in whom drugs associated with late morbidity such as anthracyclines may be reduced and conversely a higher risk group in whom further intensification of treatment would be justified.


British Journal of Cancer (1995) 72, 752-756

© 1995 Stockton Press All rights reserved 0007-0920/95 $12.00

### Childhood T-cell lymphoblastic lymphoma – does early resolution of mediastinal mass predict for final outcome?

SF Shepherd[1], RP A'Hern[2] and CR Pinkerton[3] on behalf of the United Kingdom Children's Cancer Study Group (UKCCSG)

[1]*Departments of Clinical Oncology*, [2]*Statistics and* [3]*Paediatric Oncology, Royal Marsden Hospital, Downs Road, Sutton, Surrey SM2 5PT, UK.*

**Summary** This study presents a retrospective review of chest radiography in children with Murphy stage III T-cell lymphoblastic lymphoma. All received a standard leukaemia-based protocol with intensive induction, consolidation and continuing chemotherapy. Neither initial thoracic disease bulk nor the presence of a pleural effusion predicted outcome. However a significant difference was found when the 50 patients in whom the chest radiograph returned to normal within 60 days of commencing treatment were compared with the 18 patients with persistent mediastinal abnormalities, for both event-free [hazard ratio $\leqslant$ 60 days to $>$60 days (HR) 3.55 (95% CI 1.33–9.48); $P = 0.007$] and overall survival [HR 2.95 (95% CI 1.07–8.18); $P = 0.03$]. It appears that this relatively simple estimate of chemosensitivity may identify a group of particularly good-risk patients in whom drugs associated with late morbidity such as anthracyclines may be reduced and conversely a higher risk group in whom further intensification of treatment would be justified.

**Keywords:** childhood T-cell lymphoblastic lymphoma; chest radiography; resolution of mediastinal mass

Despite a steady improvement in the results of treatment of childhood T-cell lymphoblastic lymphoma over the past 20 years, overall 30–40% of patients still relapse. Ideally, one would like to be able to predict which subgroup will have a poor outcome either before treatment or shortly after standard therapy has started. Early recognition of these children would allow the use of a more intensive treatment protocol introducing different drugs and perhaps high-dose chemotherapy with bone marrow rescue, or addition of local treatment such as mediastinal irradiation.

It is now established that T-cell lymphoblastic lymphoma and T-cell acute lymphoblastic leukaemia (T-ALL) are different parts of a disease spectrum, the latter diagnosis being defined arbitrarily when the bone marrow contains more than 25% lymphoblasts. With the exception of advanced stage at presentation, the use of patient characteristics, cell markers, immunocytochemistry and immunophenotyping have failed to provide a consistently reliable index of poor prognosis (Crist *et al*., 1988; Berger *et al*., 1990; Pui *et al*., 1990; Shuster *et al*., 1990). It remains possible that the use of high resolution cytogenetics or molecular markers may yet be able to define precise abnormalities which can be used to select treatment regimens (Lange *et al*., 1992).

The United Kingdom Children's Cancer Study Group (UKCCSG) study 8503 in advanced stage childhood T-cell lymphoblastic lymphoma (Eden *et al*., 1992) is one of the largest published using a single treatment protocol (Figure 1) for this rare malignancy. In summary, it comprised treatment induction with weekly intravenous (i.v.) vincristine for 4 weeks, daily oral prednisolone for 28 days, i.v. daunorubicin on days 1 and 2, *Erwinia* L-asparaginase intramuscularly or subcutaneously for a total of nine doses and intrathecal (i.t.) methotrexate on days 1, 15 and 28. All patients received early (week 5) and late (week 20) intensification modules consisting of i.t. methotrexate and i.v. vincristine on day 1, i.v. daunorubicin on days 1 and 2, i.v. cytosine arabinoside 12 hourly on days 1–5 inclusive, i.v. etoposide on days 1–5 inclusive, oral 6-thioguanine on days 1–5 inclusive and oral prednisolone for 7 days and tailing off by day 14 of intensification. Drugs doses are given in Figure 1.

Following recovery of blood counts after early intensification, all patients received standard treatment to the central nervous system with cranial irradiation (18 Gy) in ten fractions of 1.8 Gy over 2 weeks and three further weekly i.t. methotrexate doses at the same time. During irradiation, oral mercaptopurine was administered daily. Continuing treatment between the two intensification modules and after the second until 2 years has elapsed from when remission was achieved was with daily mercaptopurine throughout and once-weekly oral methotrexate with the addition of prednisolone orally for 5 days and a single i.v. vincristine injection every 4 weeks. All patients received oral cotrimoxazole twice daily three times a week from the beginning of week 5 until the end of all continuous therapy.

In the present study, clinical measures of disease bulk at presentation have been retrospectively collected on all the Murphy stage III patients with centrally confirmed T-cell lymphoblastic lymphoma. The objectives were to document the time to clinical and radiological complete remission, the relationship between complete remission and the presence of a pleural effusion or 'white-out', the bulk of initial disease, the size of the mediastinal mass, mediastinal mass to thorax ratio and nodal size at presentation. From these data it was hoped to determine whether or not it is possible to select prospectively which patients will do badly on pretreatment clinical criteria and on response to treatment.

### Methods

Registration data from the UKCCSG T-cell lymphoblastic lymphoma study protocol 8503 were obtained. This provided the patients' trial number, their first and surname, the centre which entered and treated them, the date of diagnosis, their clinical stage at presentation and the date they were last reviewed along with their clinical status at that time. Of the 99 patients registered, 15 were excluded from this analysis as being other than stage III (two with stage II and 13 with stage IV disease).

Original histological review had been undertaken centrally by a panel of three pathologists who used the Kiel classification system. Histology reports on the stage III patients recorded 78 patients with T-cell lymphoblastic lymphoma, five patients with large-cell anaplastic lymphoma and one patient with pleomorphic T-cell non-Hodgkin's lymphoma (NHL). To ensure a homogeneous patient population

Correspondence: CR Pinkerton
Received 8 December 1994; revised 27 March 1995; accepted 11 April 1995

**Figure 1** Outline of UKCCSG 8503 regimen. There is a standard four-drug induction followed by two intensification blocks and continuing chemotherapy up to a total of 2 years. Central nervous system-directed therapy is with intrathecal methotrexate and cranial irradiation (18 Gy).

only those with confirmed T-cell lymphoblastic lymphoma were included in this analysis.

A clinical data sheet was compiled which detailed the information required. Assessment of measurable disease was requested on day 1, between days 2 and 27 when available as assessment during this period was discretionary, on day 28 (a standard reassessment time of the 8503 regimen) and subsequently. The data collected comprised details of the presence or not of a pleural effusion, the size of any mediastinal mass and whether this was associated with a radiological 'white-out', mass to thorax ratio and the size of any palpable lymphadenopathy. The time in days from diagnosis and the start of treatment to complete radiological remission as determined by plain posteroanterior (PA) chest radiograph (CXR) was recorded. Data were taken from the original report by the radiologist, or if information was inadequate the relevant CXRs were reviewed locally. Blind central review was not performed. Computerised axial tomographic (CAT) scanning of the thorax was used neither routinely during initial staging nor to further assess residual mediastinal masses. The same applied to gallium scanning. None of the patients with persisting mediastinal abnormalities underwent biopsies to try to confirm the presence or otherwise of residual lymphoma. Lactate dehydrogenase levels were not routinely estimated during initial staging.

A total of 18 centres throughout the United Kingdom were contacted. Thirteen provided data through the post, while the other five were visited and the relevant information extracted from notes and radiographs.

For the purpose of event-free survival, patients with persistent mediastinal abnormality were considered to be in a state of relapse at 60 days. Survival is defined as time to death from any cause. For surviving patients, survival is censored at the date of last follow-up. Survival curves were constructed by the Kaplan-Meier method, 95% confidence intervals (CIs) being calculated from the asymptotic variance of log [−log(survival fraction)]. Survival curves were compared using the log-rank test, and where categories were ordered, e.g. size of lymphadenopathy, a trend test was employed. Hazard ratios (HRs) were calculated using Cox's regression (BMDP program 2L).

### Results

Clinical and radiological data were collected on all 78 stage III patients with confirmed T-cell lymphoblastic lymphoma. There was no statistically significant relationship between survival and the size of mediastinal mass, the mass to thorax ratio, a 'white-out', a pleural effusion, or lymph node disease bulk at presentation (Table I). One patient was noted to have hepatosplenomegaly and one other bulky renal disease.

Normalisation of a plain PA CXR was a measure of clinical and radiological complete remission in all but two patients, each of whom had presented with bulky lymph node masses greater than 5 cm in diameter. Both these patients remain alive and disease free.

At the time of data analysis 60 patients remained alive and well with no evidence of recurrent disease, one having been successfully salvaged after relapse. Eighteen patients had died, 14 from disease, two from infection and two from other causes. Median follow-up of living patients was 70 months, range 51–109 months.

One centre administered mediastinal irradiation routinely at the same time as cranial prophylaxis, although this was not part of the UKCCSG protocol. Seven patients were treated to a mid-plane dose of 15 Gy in ten daily fractions of 1.5 Gy over 2 weeks and their mediastinal masses resolved within 10–35 days. One patient subsequently relapsed in bone marrow and died. The other six remain alive and well. Although the mediastinal radiotherapy was given during weeks 9 and 10 of the protocol, which was at least 25 days after the resolution of the mediastinal masses of all the recipients, these patients have been excluded from the event-free and overall survival calculations. This ensured that the analysis included only those patients who received uniform treatment according to protocol.

Survival analyses were undertaken at 30, 60 and 90 days from the start of treatment. When the outcome in the 50 children whose CXRs had normalised within 60 days was compared with the outcome in the 18 in whom mediastinal abnormality persisted beyond 60 days, both 5 year event-free survival [84% (95% CI 71–92%) *vs* 56% (95% CI 31–75%); HR 3.55 (95% CI 1.33–9.48); $P = 0.007$] and 5 year overall

**Table I** Outcome in relation to initial radiological features

| *Feature* | *Number of patients* | *Survivors NED* | *Five year survival (%) (95% CI)* | P-*value* |
|---|---|---|---|---|
| Total | 78 | 60 | | |
| Mediastinal mass | 73 | 55 | | |
| No mass | 5 | 5 | 100 | |
| Mass <10 cm | 21 | 17 | 81 (57–92) | |
| Mass ⩾ 10 cm | 33 | 23 | 70 (51–82) | NS |
| 'White-out' | 19 | 15 | 79 (53–91) | |
| Mass–thorax ratio ⩽ 33% | 11 | 10 | 91 (51–99) | |
| Mass–thorax ratio >33% | 43 | 30 | 70 (54–81) | NS |
| Effusion | 43 | 35 | | |
| No effusion | 35 | 25 | 71 (53–83) | |
| Unilateral | 38 | 30 | 79 (62–89) | NS |
| Bilateral | 5 | 5 | 100 | |
| Lymphadenopathy | 49 | 40 | | |
| None | 29 | 20 | 69 (49–82) | |
| <2 cm | 19 | 14 | 74 (48–88) | |
| 2–5 cm | 20 | 18 | 90 (66–97) | NS |
| >5 cm | 10 | 8 | 80 (41–95) | |

survival [84% (95% CI 71–92%) *vs* 61% (95% CI 35–79%); HR 2.95 (95% CI 1.07–8.18); $P = 0.03$] were significantly better (Figures 2 and 3). There was almost no difference in the outcome between 60 and 90 days as only one of the patients who had not achieved a complete response (CR) by 60 days did so by day 90. The analysis at 30 days showed no statistical difference in outcome between the two groups (data not shown).

To see whether the 11 patients with a mass to thorax ratio of ⩽33% favourably influenced the outcome of the ⩽60 day group, we repeated the event-free survival analysis using only those patients with a mass to thorax ratio of >33% or with a white-out. The result remained significant [HR 3.65 (95% CI 1.02–13.02); $P = 0.03$].

In the group of long-term survivors, only 7 of the 60 had a persistent mediastinal mass 2 months from the start of treatment. Of the 14 patients who died from disease, in four an abnormal mediastinum persisted until death (at 4½, 8, 11, and 17 months from diagnosis), and in a fifth an effusion never cleared before death 14 months after presentation. One other patient who died from disease had an abnormal mediastinal outline on PA CXR for over 5 months.

The pattern of disease at relapse and outcome in the treatment failures are shown in Table II. Three out of seven rapid and five out of eight slow responders failed at the primary site of mediastinal bulk disease.

**Figure 2** Event-free survival by response at 60 days. (———, CR, $n = 50$ cases; ----, no CR, $n = 18$ cases). Difference is significant ($\chi^2 = 7.20$, d.f. = 1, $P < 0.01$).

**Figure 3** Overall survival by response at 60 days. Survival in relation to radiological complete response. Sixty-eight patients were analysed. The seven patients who received mediastinal irradiation and the two who died from infection before 60 days had elapsed from commencing chemotherapy were excluded. There was insufficient data on one patient to define radiological response. (———, CR $n = 50$ cases, ----, no CR $n = 18$ cases). Difference is significant ($\chi^2 = 4.76$, d.f. = 1, $P < 0.05$).

### Discussion

The UKCCSG T-cell NHL 8503 study used a uniform chemotherapy protocol and cranial irradiation throughout its duration, and included the majority of cases of childhood T-cell lymphoblastic lymphoma in the United Kingdom between 1985 and 1990. This study therefore provides an opportunity to examine, in an unselected, homogeneous population, clinical and radiological measures of disease at presentation and to test for any relationship between such measures, rapid initial treatment response and outcome.

The patients whose mediastinal masses resolved within 60 days of commencing chemotherapy have a significantly better survival. The decision to analyse at 30, 60 and 90 days was based on day 28 being a standard point of assessment in the 8503 protocol and our observation that the great majority of rapid responders had achieved a radiological complete response by 60 days, borne out by the 90 day result being almost the same. It is possible that with a larger prospective study a different cut-off might be obtained.

Early resolution of a mediastinal mass in this disease reflects chemosensitivity and residual abnormalities are likely to be due to persisting tumour, although thrombosis and fibrosis have been documented. T-cell lymphoblastic lymphoma is unlikely to have a significant stromal component, which is a frequent cause of a residual mediastinal abnormality in Hodgkin's disease (North *et al*., 1987; Sandrock *et al*., 1992).

Table II Site of relapse and outcome in relation to complete response (CR) as indicated by chest radiography by day 60

| | | CR ⩽60 days | CR >60 days (or never) |
|---|---|---|---|
| Relapsed and died | Bone marrow | 1[a] | 1 |
| | CNS | 1 | 1 |
| | Bone marrow + CNS | 1 | – |
| | Mediastinum | 3 | 5[b] |
| | Testicular | 1 | – |
| Relapsed and salvaged | CNS | – | 1 |
| Other deaths | Right atrial thrombus | | 1 |
| | Radiation encephalopathy | | 1 |
| | Infection | | 2[c] |

[a]Excluded from survival analysis as patient received mediastinal irradiation. [b]Progression of mediastinal mass. [c]Excluded from survival analysis as died within 60 days of starting treatment.

Using the persistence of an abnormal mediastinum on plain PA CXR more than 60 days from the start of chemotherapy, we have defined a significant prognostic factor indicating a subgroup of patients who may benefit from additional treatment. By using this simple measure of treatment response, approximately 25% of children with T-cell lymphoblastic lymphoma would appear to have a poorer prognosis.

The majority of patients presenting with T-cell lymphoblastic lymphoma in childhood have stage II disease by virtue of their mediastinal mass. In the absence of bone marrow, central nervous system (CNS) and testicular involvement at presentation, the most likely site of residual disease after systemic chemotherapy is where initial disease was most bulky. This was the commonest pattern of relapse or failure observed, with eight patients suffering recurrent or persistent mediastinal disease. Distant failure was experienced by seven children and comprised one testicular, two marrow, three CNS and one combined marrow and CNS.

There was no specified salvage therapy as part of the 8503 protocol, and the few patients who were salvaged relapsed more than 2 years out from initial treatment and underwent autologous bone marrow transplantation. This indicates the need to consider intensive salvage regimens using marrow transplant procedures to be part of future protocols, and reinforces the message of treatment intensification for any poor prognosis subgroups that can be defined prospectively.

Currently, the trend in paediatric oncology is to avoid radiotherapy wherever possible because of its side-effects on growth. However, it is conceivable that children with slow-responding T-cell lymphoblastic lymphoma mediastinal masses could have their chance of long-term survival improved by local irradiation. A previous UKCCSG study in which mediastinal radiotherapy showed a survival advantage (Mott *et al*., 1984) has been criticised because the chemotherapy regimen used was considered suboptimal, and it was suggested that the radiotherapy was compensating for this inadequacy. Nevertheless, it emphasises that control of mediastinal bulk disease is necessary for long-term survival and that, in those patients who respond slowly and/or partially to more intensive drug regimens, mediastinal irradiation could have a role. This view has been expressed by others who have reported small studies but used mediastinal radiotherapy for patients who presented with obstructive symptoms and/or responded slowly (Weinstein *et al*., 1983; Camitta *et al*., 1985). One of the arguments against mediastinal irradiation has been the unnecessary exposure of the 70% or more patients who will be cured by chemotherapy alone. Selection on the basis of initial response would avoid its use in the majority of patients.

An alternative approach to try to improve the chance of cure would be the use of more intensive chemotherapy at an early stage, again using radiological speed of response as a selection method. Exactly what this should comprise would require careful consideration, as even in the 'poor-risk' group the survival is approximately 60%. Treatment with significant early and late morbidity such as high-dose therapy with marrow rescue or combining further anthracyclines and mediastinal irradiation are unjustified without a worthwhile gain in cure.

Clearly this retrospective analysis has limitations, as by definition any data obtained in this way can only be safely used to generate a hypothesis which then has to be tested prospectively. To try to improve the discrimination using speed of response of mediastinal disease in these patients, it is planned to incorporate a series of carefully timed PA CXRs into a future study. In addition, thoracic computerised axial tomography will be performed, in order to try to improve the accuracy of assessing treatment response and to evaluate the feasibility of guided fine-needle aspirates to look for residual disease. The results will enable us to investigate further the potential of this possible prognostic factor.

In conclusion, clinical and radiological parameters on all 78 stage III patients with confirmed T-cell lymphoblastic lymphoma who were treated on UKCCSG study 8503 have been collected and analysed retrospectively. A significant 5 year event-free and overall survival benefit have been demonstrated for those patients whose mediastinal masses completely resolved by 60 days from the start of treatment using plain PA CXR assessment. This simple measure of response to chemotherapy may prove to be of use in modifying treatment early on in patients who have persistent mediastinal abnormalities, in an attempt to improve the cure rate in this rare paediatric malignancy.

### References

BERGER R, LE CONIAT M, VECCHIONE D, DERRE J AND CHEN SJ. (1990). Cytogenetic studies of 44 T-cell acute lymphoblastic leukaemias. *Cancer Genet. Cytogenet.*, **44**, 69–75.

CAMITTA BM, LAUER SJ, CASPER JT, KIRCHNER PA, KUN LE, OECHLER HW AND ADAIR SE. (1985). Effectiveness of a six-drug regimen (APO) without local irradiation for treatment of mediastinal lymphoblastic lymphoma in children. *Cancer*, **56**, 738–741.

CRIST WM, SHUSTER JJ, FALLETTA J, PULLEN DJ, BERARD CW, VIETTI TJ, ALVARADO CS, ROPER MA, PRASTHOFER E AND GROSSI CE. (1988). Clinical features and outcome in childhood T-cell leukaemia–lymphoma according to stage of thymocyte differentiation: a Pediatric Oncology Group study. *Blood*, **72**, 1891–1897.

EDEN OB, HANN I, IMESON J, COTTERILL S, GERRARD M AND PINKERTON CR. (1992). Treatment of advanced stage T-cell lymphoblastic lymphoma: results of the United Kingdom Children's Cancer Study Group (UKCCSG) Protocol 8503. *Br. J. Haematol.*, **82**, 310-316.

LANGE BJ, RAIMONDI SC, HEEREMA N, NOWELL PC, MINOWADA J, STEINHERZ PE, ARENSON EB, O'CONNOR R AND SANTOLI D. (1992). Paediatric leukaemia lymphoma with t(8;14) (q24;q11). *Leukaemia*, **6**, 613-618.

MOTT MG, CHESSELLS JM, WILLOUGHBY MLN, MANN JR, MORRIS-JONES PH, MALPAS JS AND PALMER MK. (1984). Adjuvant low dose radiation in childhood T-cell leukaemia lymphoma – report from the United Kingdom Children's Cancer Study Group (UKCCSG). *Br. J. Cancer*, **50**, 457-462.

NORTH LB, FULLER LM, SULLIVAN-HALLEY JA AND HAGEMEISTER FB. (1987). Regression of mediastinal Hodgkin's disease after therapy: evaluation of time interval. *Radiology*, **164**, 599-602.

PUI C-H, BEHM FG, SINGH B, SCHELL MJ, WILLIAMS DL, RIVERA GK, KALWINSKY DK, SANDLUND JT, CRIST WM AND RAIMONDI SC. (1990). Heterogeneity of presenting features and their relation to treatment outcome in 120 children with T-cell acute lymphoblastic leukemia. *Blood*, **75**, 174-179.

SANDROCK B, DELAAT C AND WELLS R. (1992). Significance of persistent radiographic mediastinal abnormality in pediatric Hodgkin's disease and non-Hodgkin's lymphoma (abstract). Proceedings of the Annual Meeting of the American Society of Clinical Oncology, 11: A1169.

SHUSTER JJ, FALLETTA JM, PULLEN DJ, CRIST WM, HUMPHREY GB, DOWELL BL, WHARAM MD AND BOROWITZ M. (1990). Prognostic factors in childhood T-cell acute lymphoblastic leukemia: a Pediatric Oncology Group study. *Blood*, **75**, 166-173.

WEINSTEIN HJ, CASSADY R AND LEVERY R. (1983). Long term results of the APO protocol for treatment of mediastinal lymphoblastic lymphoma. *J. Clin. Oncol.*, **1**, 537-540.